# Depiction of mental illness and psychiatry in popular video games over the last 20 years

**DOI:** 10.3389/fpsyt.2022.967992

**Published:** 2022-08-15

**Authors:** Jozef Buday, Miroslav Neumann, Jana Heidingerová, Jirí Michalec, Gabriela Podgorná, Tadeáš Mareš, Marek Pol, Jakub Mahrík, Stanislava Vranková, Lucie Kališová, Martin Anders

**Affiliations:** ^1^Department of Psychiatry, First Faculty of Medicine of Charles University and General University Hospital, Prague, Czechia; ^2^Grammar School Bilikova, Bratislava, Slovakia; ^3^Faculty Hospital Královské Vinohrady, Prague, Czechia; ^4^Institute of Clinical and Experimental Medicine, Prague, Czechia; ^5^Institute of Normal and Pathological Physiology, Center of Experimental Medicine, Slovak Academy of Sciences, Bratislava, Slovakia

**Keywords:** mental illness, video game, psychiatry-history, psychiatric care, stigma

## Abstract

Video games represent a rapidly growing media form that is a daily activity for many youths. So far, only a little attention has been paid to the portrayal of mental illnesses and psychiatric intervention within them. In our research, we explored the best-selling video games released between 2002–2021 in order to analyse these representations. We came to the conclusion that approximately 1 out of 10 popular games attempts to portray symptoms of mental illness – with a majority of 75% of them in a negative and stereotypical way. Despite the majority of mental illness depiction in popular video games being negative, there are mounting reports that certain representations have a positive impact on their player bases. Further studies are required, as to how much videogames influence the player's attitude toward this topic.

## Introduction

In recent years, a lot of focus has been placed on the stigmatizing (1) representation of mental illness (MI) and psychiatric care in mass media (2), especially in movies (3, 4), however, little to no focus has been placed on their representation in video games. The videogame industry is a rapidly expanding market – there were 2.69 billion gamers in 2020 with a projected rise to 3.07 billion in 2023 (5). Video games have a tendency to replace other media forms such as television especially in the youth population, and thus serve as an important source of information (6). While there has been some research on the positive and negative sides of gaming in relation to mental health (7, 8), only a handful of studies, covering a relatively small timeframe have thus far explored what kind of messages popular video games convey about mental illnesses and psychiatry in general to their audiences (9–11).

### Study aims

The aim of this research is to provide a comprehensive look at the most popular video games released over the last 20 years in terms of mental illness portrayal – its frequency, type and form of depiction. We have also analyzed the portrayal of psychiatric/psychological treatment depicted in these video games.

## Methods

**Figure 1:**
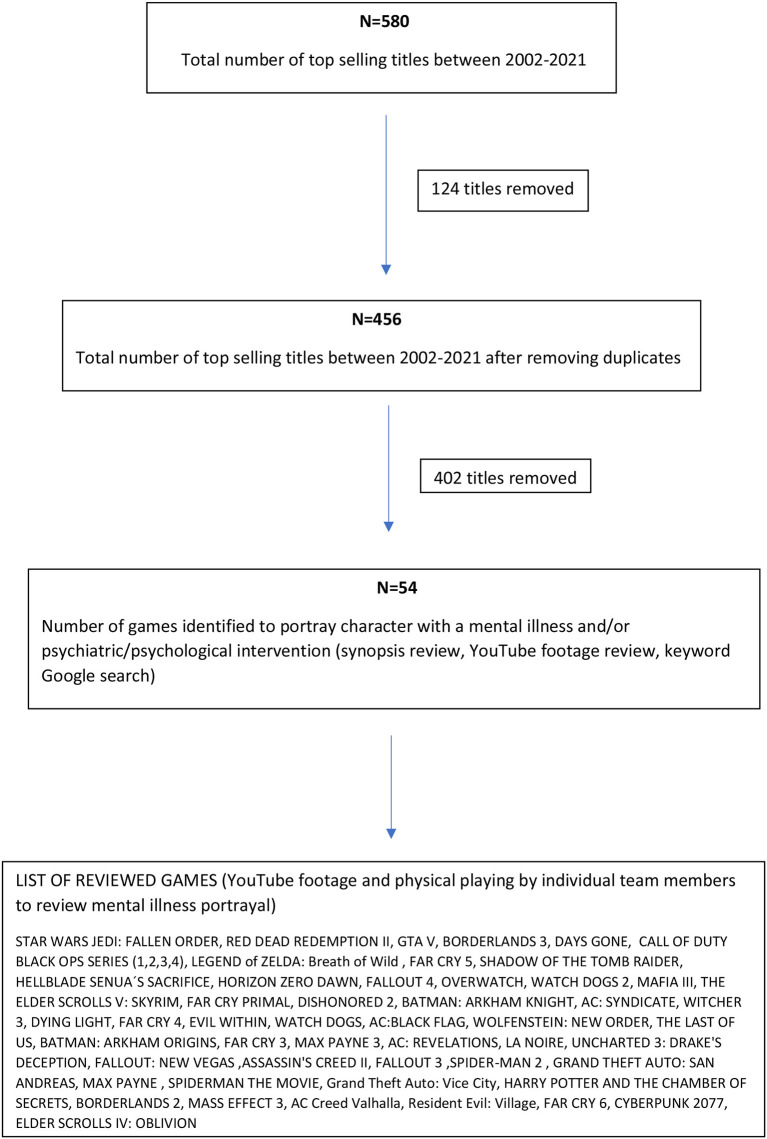
Selection of reviewed game titles.

For the purposes of this research, we utilized the statistical data of the best-selling games in the United Kingdom – UK Games Charts, that are released and made publicly available by a trade association for the UK's games and interactive entertainment industry – Ukie, for the years 2002 to 2019 (12). The data on Ukie was not available for 2020 to 2021, so we opted for the top game sales data in the UK from the Interactive Software Federation of Europe (13) (see Figure 1). We selected the top 30 best-selling games for each year from 2002 to 2019. For 2020 to 2021, we selected the top 20 titles (more data was not publicly available for the best-selling games in UK by the Interactive Software Federation of Europe) which amounted to 580 titles in total. Some games remain best-sellers for multiple years, and thus after removing duplicate titles, we ended up reviewing 456 individual titles.

These titles were screened for the presence of any portrayal of mental illness or psychiatric care/treatment – this was done with a similar strategy to Shapiro et al. (11) and Ferarri et al. (10) – we selected both formal medical terminology and colloquial/stigmatizing keyword terms, which presence we first surveyed on each game or game series' respective Wiki webpages – collaborative encyclopedias to which users provide and edit content pertaining to video games that include descriptions of characters, settings, game mechanics and narrative. Secondly, we have applied these keywords in combination with the individual game title on the Google search engine and surveyed the first 50 results.

We used search terms already utilized in the study by Shapiro et al. and added additional terms that referred to mental illness, a total of 17 keywords (“Psychotic,” “Schizophrenic,” “Depressed,” “Anxious,” “Bipolar,” “Paranoid,” “Crazy,” “Psychopath,” “Schizophrenia,” “Depression,” “Delusional,” “Manic,” “Mental Illness,” “Insane,” “Crazy Characters,” “Mentally Ill Characters,” “Insane Characters”). We excluded games that did not yield any results for these keywords or where the keywords appeared, but not in the context of a mental illness (for example, a Google search with the combination of “Crazy” and “FIFA 20” would yield links to Youtube videos titled “Crazy moments in FIFA 20”).

When we identified a game having a clear intent of a mental illness portrayal (or depiction of a psychiatric and/or psychological intervention), we have extracted the information about that game from its respective Wiki webpage and/or from a Google search link that yielded information about said depiction into an electronic database in an Excel file for further analysis. We used a general inductive approach as described by Thomas (14) and manual coding. The extracted data was thoroughly read and subsequently categorized by the type of mental illness presented. After reducing overlap and redundancy, we ended up with a total of 7 categories- schizophrenia-like illness, hallucinosis, dissociative identity disorder, depression, alcoholism, autism, other personality disorder. We further explored whether these characters were represented in a positive, neutral or negative manner. Psychological/psychiatric interventions were also categorized by type – character placed in an asylum-like facility, character undergoes psychotherapy, character undergoes a biological intervention. As was the case with the representation of MI, we analyzed whether these representations had a negative, neutral or positive impact on the character affected by them.

The selected video games were physically played by individuals on our research team (JB, GP, TM, MP, JM), so that instances that were identified to contain representation of a mental illness could be directly witnessed by a mental health professional, in order to confirm the presence of mental illness depiction gained from the keyword search. Youtube footage search was also conducted to confirm these depictions, however, it was not always possible to find a relevant result, especially with older titles – which is why we found it important to play the games directly. A personal computer (PC) was used to play the majority of games in the selected list with a few exceptions (Playstation 4 in Red Dead Redemption II, Playstation 3 – Uncharted 3, Legend of Zelda – Nintendo Switch).

### Study limitations

For the purposes of this research, we mostly utilized the UK Games Charts, which represent the most popular games sold in the United Kingdom. Although the most popular games will find their way into the top 30 in other countries as well, it is likely that if one were to use the best-selling charts in Northern America, Europe, South America or Asia, the content of that list might be slightly varied due to different local preferences. Another weakness of this study is the fact that we have used the top 30 sold games of each year (top 20 for the years 2020 and 2021), and thus it is possible that some games that depict mental illness or psychiatric care have not reached the list in our research.

Finally, we wish to add that some reviewed games were identified on their Wiki pages to contain metaphorical representations of mental illnesses, however, did not explicitly portray symptoms of a mental illness (for instance, a character in Kingdom Hearts III is referring to be fighting “darkness” which several Google engine sources claim to be a metaphorical representation of depression, however, in the medical sense, she is not manifesting any symptoms of depression)– these games were thus not included as a graphic presentation of a mental illness.

## Results

From the 456 games reviewed, 54 games included a representation of a mental illness or its symptoms, which amounts to 12%. Some games portrayed various mental illnesses. All in all, we recorded a total of 57 instances of MI portrayal in these games. 43 instances were negative (75%), 13 were neutral and only 1 instance was clearly depicted as positive.

**Figure 2:**
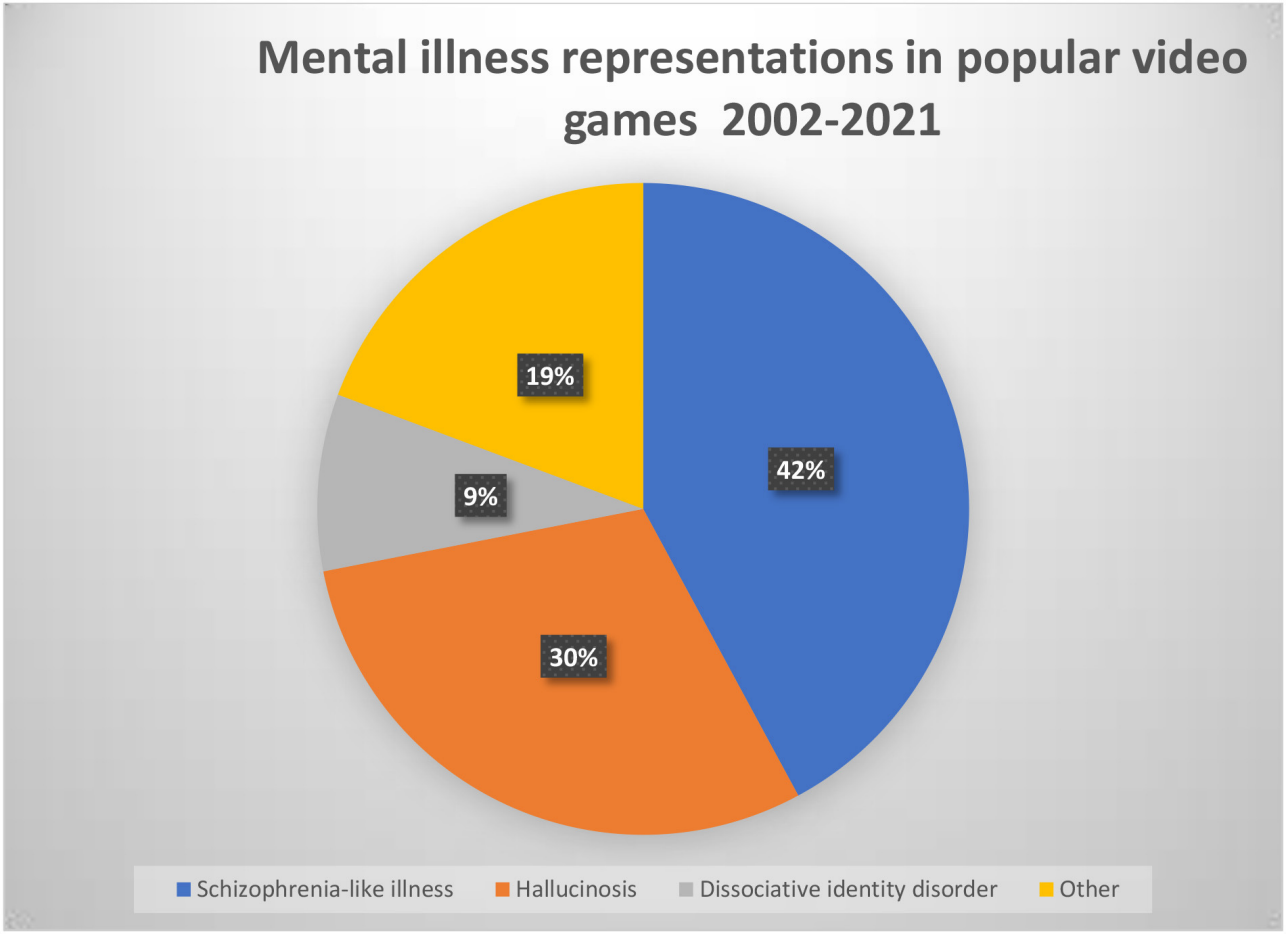
Distribution of the type of mental illnesses commonly portrayed in popular video games. Total number of instances *N* = 57.

The most popular type of portrayed MI was a schizophrenia-like illness (see Figure 2), which was sometimes represented within multiple characters (in a single game) in a total of 24 games. An overwhelming majority – 20 of these video games represented schizophrenic illness in a negative manner – in nearly all of these instances, characters are depicted as violent, homicidal, suffering from either paranoid or megalomanic delusions and audiovisual hallucinations. In most cases, these characters are killed by the player character (see Table 1). Only 4 of the reviewed video games represented this illness in a more balanced/neutral way.

**Table 1:** Individual video game titles and a brief summary of the portrayed mental illness or depicted intervention.

**Game**	**Symptoms/mental Illness portrayal**	**Positive/neutral/negative**	**Negative outcome for the characters present?**	**Is any psychiatric/psychological intervention described?**
Star wars jedi: fallen order	Schizophrenia-like illness - character Talon Malicos is described as descending into madness, suffering from megalomanic delusions induced by a supernatural concept	Negative	The character is killed	No
Red dead redemption ii	Schizophrenia-like illness - at least 3 instances of “mad” or violent characters driven by paranoid or megalomanic delusions	Negative	The characters are killed	No
Gta v	Several instances of toxic psychosis and a violent main character (Trevor) with an unspecified, but mentioned personality disorder with violent homicidal rants	Negative	No	Psychoanalysis-like sessions with a cynical, uncaring and overcharging psychiatrist who can be killed by the main protagonist
Borderlands 3	Dissociative identity disorder - present in one of the main game characters (Krieg the “Psycho”) - one of the personalities is homicidal and violent	Negative	No	No
Days gone	Schizophrenia-like illness in one of the antagonists with megalomanic delusion and one side character with a delusional disorder that acts violently/homicidal toward those who disagree with his conspiracy theories	Negative	No	No
Call of duty black ops series (1,2,3,4)	Toxic psychosis - several instances of hallucinations induced by an unknown psychoactive substance - reference to the MK-ultra program conducted by the American secret service	Neutral	No	No
LEGEND of ZELDA: Breath of Wild	Schizophrenia-like illness in 2 side characters clearly depicted as insane and can turn violent toward the player	Negative	The characters can be killed by the player	No
Far cry 5	Several instances of toxic psychosis, violent main character suffering from a schizophrenia-like illness with megalomanic delusions	Negative	The character brings on an apocalypse by detonating what seems like a nuclear bomb	Elements of cbt are used by the antagonists to torture victims into complacency
Shadow of the tomb raider	Toxic psychosis induced by an unknown psychedelic substance	Negative	No	No
Hellblade senua's sacrifice	The main character is suffering from a schizophrenia-like illness with various types of audiovisual hallucinations	Neutral	No	No
Horizon zero dawn	A side character is suffering from a schizophrenia-like illness that resulted in homicide due to violent auditive hallucinations	Negative	The character with schizophrenia ends up in self-imposed isolation	No
Fallout 4	Toxic psychosis and multiple instances of characters suffering from a schizophrenia-like illness resulting in violent behavior and homicides	Negative	Characters with schizophrenia are killed by the player	No
Overwatch	One of the playable characters suffers from a schizophrenia-like illness induced by “exposure to a black hole”.	Negative	No	Treatment by restraints/sedation in a fictional asylum.
Watch dogs 2	One of the main characters is diagnosed with a form of highly functional autism	Positive	No	No
Mafia iii	The main character is suffering from a PTSD like illness experiencing flashbacks	Negative	No	No
The elder scrolls v: skyrim	One of the main characters in the game is suffering from a schizophrenia-like illness with personality change, audiovisual hallucinations and homicidal behavior	Negative	The player can choose to kill the character	No
Far cry primal	Toxic psychosis	Negative	No	No
Dishonored 2	Dissociative identity disorder - one of the main antagonists has a violent and homicidal personality	Negative	The player can choose to kill the character	No
Batman: arkham knight	Toxic psychosis	Negative	No	No
Ac: syndicate	Instances of schizophrenia-like illness - patients represented as disoriented experimentation subjects	Negative	Yes - deaths and experimentation	Ect and psychiatric care described as inhumane, experimental and torturous
Witcher 3	Depression and alcoholism, schizophrenia-like illness, catatonia	Neutral	Depending on the player's choices	The player can intervene and help one of the main protagonists suffering from depression
Dying light	A side character is suffering from a severe development disorder, oblivious to the apocalyptic events around him	Neutral	No	No
Far cry 4	Toxic psychosis	Negative	No	No
Evil within	Schizophrenia-like illness induced by supernatural entity	Negative	Yes - deaths	Psychiatric asylum with straightjackets as a routine method of treatment
Watch dogs	A character in the game is diagnosed with severe PTSD after witnessing the death of his mother	Neutral	No	The character is treated by a psychologist
Ac:black flag	Schizophrenia like illness in one of the characters	Neutral	No	No
Wolfenstein: new order	A group of patients with a severe mental illness is murdered by a group of German soldiers	Negative	Yes - deaths	The patients are originally treated in a polish asylum where a psychiatrist and a nurse treat them well
The last of us	Schizophrenia like illness in one of the characters	Neutral	No	No
Batman: arkham origins	Severe personality disorders usually resulting in violent and homicidal behavior	Negative	Yes - violence, imprisonment	An asylum represented as a prison
Far cry 3	Toxics psychosis and a main character with antisocial personality disorder	Negative	Yes - death	No
Max payne 3	Trauma induced schizophrenia-like illness of the main character	Negative	Yes - alcohol/painkiller abuse	No
Ac: revelations	Hallucinosis	Neutral	No	No
La noire	At least 3 instances of suspected suffering from a schizophrenia-like illness resulting in homicides	Negative	Yes - death, imprisonment	Mention of an asylum for the criminally insane
Uncharted 3: drake's deception	Toxic psychosis	Neutral	No	No
Fallout: new vegas	The player explores a vault which residents have been experimented upon in various ways in order to induce a schizophrenia-like illness in them with paranoid delusions	Negative	Yes - the residents died	No
Assassin's creed ii	Hallucinosis	Neutral	No	No
Fallout 3	Multiple instances of a schizophrenia-like illness resulting in homicides	Negative	Yes - death	No
Spider-man 2	Dissociative personality disorder - one of the personalities of the antagonist is described as homicidal and violent	Negative	No	No
Grand theft auto: san andreas	Depression and alcoholism with suicide attempts	Neutral	No	The player intervenes and the depressed patient is placed in a “rehab clinic”
Max payne	Trauma induced schizophrenia-like illness of the main character resulting in horror like hallucinations and scary auditive hallucinations	Negative	Yes - alcohol/painkiller abuse	No
Spiderman the movie	Dissociative identity disorder - one of the personalities of the antagonist is described as homicidal and violent	Negative	No	No
Grand theft auto: vice city	Schizophrenia like illness in one of the characters	Negative	Yes - the character is killed by the player	No
Harry potter and the chamber of secrets	Narcissistic personality disorder in one of the characters	Negative	Yes - the character ends up “descending into madness”	The character is placed in a magical asylum for the insane, seemingly without hope for efficient treatment
Borderlands 2	Dissociative identity disorder - present in one of the main game characters (Krieg the “Psycho”) - one of the personalities is homicidal and violent	Negative	No	No
Mass effect 3	A character encountered in the game is being treated for PTSD	Neutral	Yes - depending on the player's choices, the character can be killed	The character is treated via unspecified psychotherapy at a hospital
Ac creed: valhalla	King Charles is suffering from a schizophrenia-like illness resulting in homicides	Negative	Yes - death	No
Resident evil village	Hallucinosis induced by a supernatural entity	Negative	No	No
Far cry 6	Toxic psychosis	Neutral	No	No
Cyberpunk 2077	Schizophrenia-like illness called “cyberpsychosis” resulting in paranoid delusions and violent behavior on multiple variations	Negative	Yes -depending on the player's choices, the characters die	No
Elder scrolls iv: oblivion	Schizophrenia-like illness in multiple characters resulting in homicide	Negative	Yes - death	No

The second most popular type of portrayed MI was hallucinosis, usually induced by a psychoactive substance, or, in some cases – a supernatural entity. Hallucinosis was represented in 17 video games – in all cases, they are represented as audiovisual and the majority of them are portrayed as horror-like or otherwise fear-inducing.

A personality disorder was recorded in 8 video games, the most popular type (5 instances) was dissociative identity disorder. In all cases, it was represented as the character having a violent, dangerous, unpredictable and homicidal alter ego.

Other mental illnesses were rather rare. In contrast to psychotic illnesses, depression with alcohol addiction and suicide risk were portrayed in only 2 instances in a neutral manner.

The only clearly positive depiction of a character was represented in Watch Dogs 2 – one of the characters was diagnosed with a highly functional type of autism.

From the 456 games reviewed, 13 games included a portrayal of a form of psychiatric or psychological intervention – 3%. 8 out of these games depicted this intervention as negative, the remaining 5 games portrayed this intervention in a neutral way or with an unspecific outcome for the character. No video game included in our list portrayed a form of this type of intervention in a positive manner.

## Discussion

Depiction of a psychotic schizophrenia-like illness is the most prevalent representation of a mental illness in video games. The characters suffering from this type of illness are usually represented as dangerous, violent and homicidal. Some notable depictions include.

*The Elder Scrolls IV: Oblivion (2006)* contains two representations of a psychotic schizophrenia-like illness. In the quest “Paranoia,” the player is approached by an elf named Glarthir, who mentions his fears of being followed by various people in town. The player is then tasked to spy on these people, eventually discovering that none of them are actually following Glarthir, making it clear that the elf is suffering from paranoid delusions. Upon delivering the information to him, the elf violently assaults the player (and ends up being killed), who then has a dialogue option to conclude that Glarthir was crazy. During the “Following a Lead” quest, the player meets Mathieu Bellamont, a violent assassin character who is depicted as having a trauma-induced schizophrenia-like illness after witnessing the murder of his mother. The character is depicted as experiencing auditive hallucinations while talking to the severed head of his mother and keeping a diary that contains incoherent and violent writings apparently done in blood. The very famous successor of Oblivion, *The Elder Scrolls V: Skyrim (2011)*, continues with this particular representation – one of the more prominent characters in the game, Cicero, is depicted as a violent, homicidal assassin who also has a trauma-induced schizophrenia-like illness – he is seen suffering from audiovisual hallucinations, paranoid delusions and personality change.

While there have been real-world instances of people suffering from psychosis committing violent crimes and murders, they only represent a very small fraction (15–17). Although some link exists between violence and the presence of a serious mental illness (18, 19), individuals with serious mental illness are victimized by violent acts more often than they commit them (20). It should be also noted that while the majority of MI representation in popular video games is unrealistic and negative, so are other aspects of most video games. In the case of the aforementioned Elder Scrolls series for instance, the player spends most of the time doing unrealistic tasks – fighting fantasy creatures such as dragons, goblins or minotaurs, casting fireballs, exploring zombie infested dungeons or summoning creatures from an alternate dimension. Most other characters are presented in a one-sided manner as either being “good” or “evil” or with extremely exaggerated personality traits. These mechanics often go unnoticed and therefore, we argue that it is entirely possible that negative representations of MI might also go unnoticed in the wider context of the videogame.

Some video games portrayed mental illness in a more balanced point of view. In the *Witcher 3: Wild Hunt (2015)* for instance, one of the most important characters in the game, the Baron, is portrayed as a man suffering from depression with alcohol abuse in a matrimonial crisis. The player can choose to intervene and help the Baron, who, depending on the player's choices can overcome these issues and reunite his family. In *Hellblade: Senua's Sacrifice (2017)*, the main character is depicted as suffering from a schizophrenia-like mental illness. She experiences various audiovisual hallucinations, not all of which are horror-inducing. The developers of the game worked with medical professionals and real-life patients diagnosed with a psychotic illness in order to recreate their experiences in an authentic manner (21, 22).

These examples are a clear indication that video games are able to relate positive messages about mental illness and present them as issues that have a solution and might be useful in reducing their stigma (7, 23). In the case of *Hellblade: Senua*”*s Sacrifice*, it was also reported that the game managed to create a safe space for conversations about mental illness (24, 25). It is worthwhile mentioning that while these two smaller-scale games did not make the list in this study, both *Depression Quest* and *Actual Sunlight* (26) were reported to stimulate similar responses within their player bases.

The depiction of psychiatric interventions is rather rare compared to depictions of mental illnesses. In most instances, however, they are depicted as either costly and ineffective, or negative light.

In *Assassin's Creed: Syndicate (2015)*, the player is tasked with a quest named “Overdose” to stop an electroconvulsive therapy session. The questline takes place in an eery-looking Victorian asylum-like facility, where stacks of sedated and disoriented patients aimlessly wander its corridors. It is implicated that the staff uses these patients for various forms of experimentation. Electroconvulsive therapy (ECT) is portrayed with two staff members delivering electrical shocks (with sparks) utilizing a visually complicated device that causes a lot of pain. The character named Dr. Elliotson, who is responsible for experiments in this facility casually says that it “fried” the brain of one of the patients a “bit too much.” He is eventually assassinated by the main character while being called a dangerous lunatic.

ECT is nowadays generally considered to be a safe and very effective biological method of treatment (27, 28), especially in pharmacologically resistant affective disorders. While it is experiencing a steady rise in its use, the full potential of this method has been “successfully” hindered by its controversial image in the eyes of the general public (4). Its negative portrayal in the movies “One Flew Over the Cuckoo's Nest” and “Requiem for a Dream” has been well documented in the past.

In *Grand Theft Auto V (2013)*, one of the main protagonists, Michael, is seen in multiple scenes visiting a psychiatrist, Dr. Friedlander, in what remotely resembles psychoanalytical sessions. The psychiatrist is portrayed as cynical, uncaring and financially overcharging – ultimately offering no real help and only vague advice for Michael's real-life problems. At the end of the game, Dr. Friendlander says that he cannot treat Michael any longer and the player has the option to kill him.

Another type of common psychotherapy, cognitive-behavioral therapy (CBT) is not excluded from stigmatization either – in *Far Cry 5 (2018)*, the theorem behind CBT is used to torture and condition characters into submitting to the will of the main antagonist.

As far as we know, there are currently only three other articles that have directed their attention toward the depiction of mental illness in video games.

Ferarri et al. (10) studied the messages about MI in video games by reviewing games released between January 2016 and June 2017 on the Steam platform. This study came to the conclusion that many of the reviewed games perpetuate negative stereotypes about mental illness, however, the research focused on a short time frame and one particular platform (Steam) – some of the games included in the research are not very popular and have only hundreds of players and therefore their reach is not extensive.

Shapiro et al. (11) analyzed depictions of mental illness across the top 50 best-selling video games each year from 2011 to 2013. They came to the conclusion that video games contain frequent and varied portrayals of mental illness, with depictions most commonly linking mental illness to dangerous and violent behaviors.

Anderson (9) published an article about 3 instances of mental illness portrayal in video games and suggested that more extensive research that would include the best-selling games is needed to assess how MI is represented in popular video games.

Finally, we would like to add that the authors are not in any way commenting on the video game quality. This research is merely an observation of how they portray mental illness or professional mental health intervention.

## Conclusion

Roughly 1 out of 10 of the most popular video games that were released in the last 20 years portray symptoms of a mental illness. 75% of this content depict characters with a mental illness in a negative way. The most common type of portrayal is a schizophrenia-like illness with paranoid delusions. In contrast, only a fraction (3%) of video games portray an attempted intervention for these symptoms - the majority of which is negative toward psychiatry and represents the medical field in a negative or ineffective manner. While now-a- days, mental-health professionals are usually aware of the negative representation of psychiatry in the movie industry, the video game field and its impact on the perception of mental illness and psychiatry has been thus far largely overlooked. Despite the majority of mental illness representation in this study being negative, there are mounting reports that certain representations have a positive impact on their player bases. Further studies are required, as to how much videogames influence the player's attitude toward this topic.

## Data availability statement

The original contributions presented in the study are included in the article/supplementary material, further inquiries can be directed to the corresponding author.

## Author contributions

MA and LK were advisors for scientific methods and data processing. SV was responsible for making the tables/figures. GP and JM were responsible for the categorization of the collected data. JB, MN, MP, JM, TM, and JH collected raw data and physically played the reviewed video games. All authors listed have made a substantial, direct, and intellectual contribution to the work and approved it for publication.

## Funding

This work was supported by MH CZ-DRO VFN 64165 and Q27/LF1; Scientific Grant Agency of The Ministry of Education, Science, Research and Sport of the Slovak Republic VEGA 2/0118/21 and Cooperatio Program, research area Neuroscience.

## Conflict of interest

The authors declare that the research was conducted in the absence of any commercial or financial relationships that could be construed as a potential conflict of interest.

## Publisher's note

All claims expressed in this article are solely those of the authors and do not necessarily represent those of their affiliated organizations, or those of the publisher, the editors and the reviewers. Any product that may be evaluated in this article, or claim that may be made by its manufacturer, is not guaranteed or endorsed by the publisher.

## References

[1] Hyler SE Gabbard GO Schneider I. Homicidal maniacs and narcissisfic parasites: stigmatization of mentally ill persons in the movies. Psychiatr Serv. (1991) 42:1044–8. 10.1176/ps.42.10.1044 1959896

[2] Wahl OF. Mass media images of mental illness: a review of the literature. J Commun Psychol. (1992) 20:343–52. 10.1002/1520-6629(199210)20:4<343::AID-JCOP2290200408>3.0.CO;2-2

[3] Owen PR. Portrayals of schizophrenia by entertainment media: a content analysis of contemporary movies. Psychiatr Serv. (2012) 63:655–9. 10.1176/appi.ps.201100371 22555313

[4] Sienaert P. Based on a True Story? The portrayal of ECT in international movies and television programs. Brain Stimul. (2016) 9:882–91. 10.1016/j.brs.2016.07.005 27522170

[5] Finances Online (2020). Available online at: https://financesonline.com/number-of-gamers-worldwide/ (accessed June 12, 2022).

[6] Bogost I. Persuasive Games: The Expressive Power of Videogames. MIT Press. (2010).

[7] Granic I Lobel A Engels RC. The benefits of playing video games. Am Psychol. (2014) 69:66. 10.1037/a0034857 24295515

[8] Lau HM Smit JH Fleming TM Riper H. Serious games for mental health: are they accessible, feasible, and effective? A systematic review and meta-analysis. Front Psychiatry. (2017) 7:209. 10.3389/fpsyt.2016.00209 28149281 PMC5241302

[9] Anderson SL. Portraying mental illness in video games: exploratory case studies for improving interactive depictions. Loading. (2020) 13:20–33. 10.7202/1071449ar

[10] Ferrari M McIlwaine SV Jordan G Shah JL Lal S Iyer SN. Gaming with stigma: analysis of messages about mental illnesses in video games. JMIR Mental Health. (2019) 6:e12418. 10.2196/12418 31066703 PMC6707601

[11] Shapiro S Rotter M. Graphic depictions: portrayals of mental illness in video games. J Forensic Sci. (2016) 61:1592–5. 10.1111/1556-4029.13214 27783392

[12] UK Games Charts, ukiepedia. (2021). Available online at: https://ukiepedia.ukie.org.uk/index.php/UK_Games_Charts (accessed June 12, 2022).

[13] UK Annual Report. (2021). Available online at: https://www.gamesindustry.biz/articles/2021-01-08-43-million-games-sold-in-the-uk-in-2020-uk-annual-report (accessed June 12, 2022).

[14] Thomas DR. A General Inductive Approach for Qualitative Data Analysis. SAGE (2003).

[15] Angermeyer MC Matschinger H. The effect of violent attacks by schizophrenic persons on the attitude of the public towards the mentally ill. Soc Sci Med. (1996) 43:1721–8. 10.1016/S0277-9536(96)00065-2 8961416

[16] Buchanan A Sint K Swanson J Rosenheck R. Correlates of future violence in people being treated for schizophrenia. Am J Psychiatry. (2019) 176:694–701. 10.1176/appi.ajp.2019.18080909 31014102

[17] Cho W Shin WS An I Bang M Cho DY Lee SH. Biological aspects of aggression and violence in schizophrenia. Clin Psychopharmacol Neurosci. (2019) 17:475–86. 10.9758/cpn.2019.17.4.475 31671484 PMC6852683

[18] Imhoff R. Zeroing in on the effect of the schizophrenia label on stigmatizing attitudes: a large-scale study. Schizophr Bull. (2016) 42:456–63. 10.1093/schbul/sbv137 26409222 PMC4753605

[19] Stuart H. Violence and mental illness: an overview. World Psychiatry. (2003) 2:121–124. 16946914 PMC1525086

[20] Thornicroft G Brohan E Rose D Sartorius N Leese M INDIGO Study Group. Global pattern of experienced and anticipated discrimination against people with schizophrenia: a cross-sectional survey. Lancet. (2009) 373:408–15. 10.1016/S0140-6736(08)61817-6 19162314

[21] Austin J. “*The Hardest Battles Are Fought in the Mind”: Representations of Mental Illness in Ninja Theory's Hellblade: Senua's Sacrifice*. Game Studies (2021).

[22] Meakin E Vaughan B Cullen C. “Understanding” Narrative; Applying Poetics to Hellblade: Senua's Sacrifice. Game Studies (2021).

[23] Ferrari M Bush N Clark D Archie S. Debris: Exploring the Video Game Values That Can Help Reduce Mental Illness Stigma. (2016). Available online at: http://www.digra.org/digital-library/publications/debris-exploring-the-videogame-values-that-can-help-reduce-mental-illness-stigma (accessed June 12, 2022).

[24] Fordham J Ball C. Framing mental health within digital games: an exploratory case study of Hellblade. JMIR Mental Health. (2019) 6:e12432. 10.2196/12432 30998224 PMC6495293

[25] Ortiz VM. The Power of Video Games: How Celeste and Hellblade Address Mental Health. CalPoly (2021).

[26] Hoffman KM. Social and cognitive affordances of two depression-themed games. Games Cult. (2019) 14:875–95. 10.1177/1555412017742307

[27] Sackeim HA. Modern electroconvulsive therapy: vastly improved yet greatly underused. JAMA Psychiatry. (2017) 74:779–80. 10.1001/jamapsychiatry.2017.1670 28658461

[28] The UK ECT Review Group. Efficacy and safety of electroconvulsive therapy in depressive disorders: a systematic review and meta-analysis. Lancet. (2003) 361:799–808. 10.1016/S0140-6736(03)12705-5 12642045

